# Heterogeneity of heterogeneities in neuronal networks

**DOI:** 10.3389/fncom.2014.00161

**Published:** 2014-12-10

**Authors:** Fabiano Baroni, Alberto Mazzoni

**Affiliations:** ^1^School of Psychological Sciences, Monash UniversityClayton, VIC, Australia; ^2^NeuroEngineering Laboratory, Department of Electrical and Electronic Engineering, University of MelbourneParkville, VIC, Australia; ^3^Centre for Neural Engineering, University of MelbourneCarlton, VIC, Australia; ^4^The BioRobotics Institute, Scuola Superiore Sant'AnnaPontedera, Italy

**Keywords:** integrate-and-fire neurons, excitation/inhibition balance, neuronal network dynamics, biophysical heterogeneity, dynamical heterogeneity, signal modulation, high-frequency oscillations

Neurons in the brain exhibit a broad spectrum of heterogeneities even within a given morphological or physiological class. In a recent modeling study, Mejias and Longtin investigated the effects of heterogeneity in the voltage threshold for spike generation on the dynamics of random networks of excitatory and inhibitory neurons (Mejias and Longtin, 2014), hence extending their previous results on purely excitatory networks (Mejias and Longtin, 2012).

The authors focused on the different effects of heterogeneity when incorporated in either the excitatory or the inhibitory population. A greater heterogeneity in the excitatory population increases the average firing rate of both neuron types, because the subset of most excitable excitatory neurons provides a positive feedback to the whole network. Conversely, when heterogeneity is included in the inhibitory population, only the average firing rate of inhibitory neurons increases, while the average firing rate of excitatory neurons decreases. This result can be explained by the presence of low-threshold, highly excitable inhibitory neurons which tend to shift the average firing rate of the inhibitory population to higher levels, while increasing inhibitory currents in the network. While the silencing effect of an increase in inhibitory currents in the heterogeneous inhibitory population is overcome, on average, by the presence of highly excitable neurons, it dominates the dynamics of the homogeneous excitatory population, hence reducing the average firing rate of excitatory neurons.

While firing rates increase with heterogeneity of excitatory cells, potentially leading to run-away excitation in the absence of saturation or adaptation mechanisms, other features of network dynamics exhibit a non-monotonic dependence on heterogeneity. For example, the encoding of an oscillatory input signal is optimal when the network exhibits an intermediate level of heterogeneity, consistently with a recent experimental study (Tripathy et al., 2013). This behavior is reminiscent of stochastic resonance, a general phenomenon observed in excitable systems, whereby intermediate levels of noise enable optimal information encoding. In fact, heterogeneity can be considered as a form of spatial noise.

When discussing about heterogeneity in the nervous system, it is important to distinguish between biophysical heterogeneity, which relates to neuronal parameters (in simulations) or quantities that are static in the time scales of interest (in experiments), and dynamical heterogeneity, which refers to measures of ongoing neuronal activity such as firing rates and correlations. The relationships between the two can be usefully explored in both directions: while Mejias and Longtin explored the dynamical consequences of different levels of biophysical heterogeneity (bottom-up), others started from experimental observations of dynamical heterogeneity, and investigated neuronal models that are consistent with the observed dynamics (top-down, Koulakov et al., 2009, Roxin et al., 2011).

Crucially, different biophysical sources of heterogeneity can yield similar effects at the level of network dynamics. For example, the strongly skewed, lognormal-like distribution of firing rates typically observed in large-scale neuronal recordings (recently reviewed in Buzsáki and Mizuseki, 2014) can be explained by models that include nonrandom connectivity among linear neurons (Koulakov et al., 2009), as well as by homogeneous networks with random connectivity among more realistic nonlinear neurons, due to the expansive nonlinearity of the *f-I* curve (that is, the superlinear increase in output firing rate *f* with increasing input current *I*) in the presence of noise (Roxin et al., 2011). Similarly, delay and synaptic time scale diversity yield equivalent effects (Biggio et al., 2013).

Further, neuronal heterogeneity can arise from different biophysical substrates, and how the effects of different sources of heterogeneity interact is unclear. In fact, heterogeneity has been observed in virtually every aspect of neuronal physiology where it has been investigated. These include intrinsic neuronal properties, which are mostly determined by ion channels' dynamics, such as neuronal excitability, propensity to bursting, amplitude and time course of spike-frequency adaptation, post-inhibitory rebound, and many more (e.g., Marder, 2011; Angelo et al., 2012); as well as properties related to the connectivity among neurons, such as dynamics and efficacy of synaptic transmission, density and size of dendritic spines, thickness and myelination of axons (e.g., Dobrunz and Stevens, 1997; Song et al., 2005; Wang et al., 2008).

Mejias and Longtin reported that, in their simulations, the effects of heterogeneity in the distance-to-threshold of excitatory and inhibitory neurons summed linearly when combined. However, we should expect that, in general, the effects of heterogeneity in different biophysical parameter might interact in a complex manner. For example, Roxin et al. reported that heterogeneity in synaptic weights can decrease the variability in firing rates caused by the expansive nonlinearity of the *f-I* curve, contrary to expectations of a linear interaction between these two sources of variability (Roxin et al., 2011). The effects of neuronal heterogeneity are expected to be more complex in more physiologically relevant settings, where different sources of heterogeneity coexist and are distributed in a highly non-random fashion (Yassin et al., 2010).

While biophysical heterogeneity is widespread at all levels of description, we believe that important insights into the relationships between biophysical and dynamical heterogeneities can be obtained using reductionist approaches, where the degree of biophysical diversity can be described by few parameters. For example, neuronal network studies that investigated the role of connectivity heterogeneity yielded important insights by focusing on random and scale-free connectivity (described by a single parameter), both of which display important dynamical differences with respect to homogeneous all-to-all connectivity. We propose that a similar approach could be fruitfully applied to other forms of biophysical heterogeneity, and ultimately result in useful taxonomies of the different sources of biophysical heterogeneity, describing the dynamical heterogeneities they result in and the interactions between their effects. This level of understanding would facilitate the conceptual integration of different results and eventually lead to basic functional principles of neuronal processing beyond area- or species- specific details.

## Conflict of interest statement

The authors declare that the research was conducted in the absence of any commercial or financial relationships that could be construed as a potential conflict of interest.
